# Efficacy of AST-120 for Patients With Chronic Kidney Disease: A Network Meta-Analysis of Randomized Controlled Trials

**DOI:** 10.3389/fphar.2021.676345

**Published:** 2021-07-26

**Authors:** Pei-Yu Su, Ya-Han Lee, Li-Na Kuo, Yen-Cheng Chen, Chiehfeng Chen, Yi-No Kang, Elizabeth H. Chang

**Affiliations:** ^1^ Department of Pharmacy, Wan Fang Hospital, Taipei Medical University, Taipei, Taiwan; ^2^ Department of Clinical Pharmacy, School of Pharmacy, Taipei Medical University, Taipei, Taiwan; ^3^ Department of Internal Medicine, Division of Nephrology, Wan Fang Hospital, Taipei Medical University, Taipei, Taiwan; ^4^ School of Medicine, College of Medicine, Taipei Medical University, Taipei, Taiwan; ^5^ Cochrane Taiwan, Taipei Medical University, Taipei, Taiwan; ^6^ Evidence-Based Medicine Center, Wan Fang Hospital, Taipei Medical University, Taipei, Taiwan; ^7^ Department of Surgery, Division of Plastic Surgery, Wan Fang Hospital, Taipei Medical University, Taipei, Taiwan; ^8^ Department of Public Health, School of Medicine, College of Medicine, Taipei Medical University, Taipei, Taiwan; ^9^ Research Center of Big Data and Meta-analysis, Wan Fang Hospital, Taipei Medical University, Taipei, Taiwan; ^10^ Institute of Health Policy and Management, College of Public Health, National Taiwan University, Taipei, Taiwan; ^11^ Research Center for Pharmacoeconomics, College of Pharmacy, Taipei Medical University, Taipei, Taiwan

**Keywords:** chronic renal failure, adsorbent, kremezin, uremic toxin, sorbent

## Abstract

AST-120, an oral spherical activated carbon, may delay the need for kidney dialysis and improve uremia symptoms because it can adsorb acidic and basic organic compounds, especially small-molecule uremic toxins. However, previous studies produced no conclusive evidence regarding the benefits of AST-120 in delaying the progression of chronic kidney disease (CKD). Therefore, this systematic review and network meta-analysis evaluated the effects of AST-120 in patients with CKD. Related keywords of CKD and AST-120 were used to search four databases to obtain potential evidence on this topic, and two authors individually completed evidence selection, data extraction, and quality assessment. Network meta-analysis was performed for mortality, end-stage renal disease, composite renal outcomes, and laboratory outcomes based on a frequentist approach. In total, 15 randomized controlled trials (*n* = 3,763) were included in the present synthesis, and the pooled results revealed non-significant differences in mortality among the treatment strategies. Low- and high-dose AST-120 were not superior to no AST-120 treatment regarding renal outcomes. However, the event rates of end-stage renal disease (risk ratio [RR] = 0.78, 95% confidence interval [CI] = 0.62–0.99) and composite renal outcomes (RR = 0.78, 95% CI: 0.63–0.97) were significantly lower in the tailored-dose AST-120 group than in no AST-120 group. The results did not reveal a small-study effect on the outcomes. Tailored dosing of AST-120 appeared to represent an optimal treatment strategy because it resulted in lower rates of composite renal outcomes and end-stage renal disease.

## Introduction

Chronic kidney disease (CKD) is a direct cause of morbidity and mortality and a risk factor for cardiovascular disease, illustrating its significant effect on global health. According to the Global Burden of Disease Study 2017, CKD accounted for 1.2 million deaths (95% uncertainty interval [UI] = 1.2–1.3), and the global prevalence of CKD was 9.1% (95% UI = 8.5–9.8). CKD, as a direct cause of impaired kidney function and cardiovascular disease, is responsible for 23.5 million disability-adjusted life-years (95% UI = 22.2–28.9) and 1.4-million cardiovascular disease-related deaths (95% UI = 1.2–1.6). (Bikbov et al., 1990). In addition, CKD progression also increases healthcare expenditures because patients with end-stage renal disease eventually require dialysis or kidney transplantation. (Schulman et al., 2014). Hence, early detection and appropriate treatment of CKD are important for both patients and clinicians in clinical practice. (Bikbov et al., 1990). Although diet control, lifestyle modification, and conventional drug treatment are used to prevent progression to end-stage renal disease or death, these strategies may not effectively control CKD. Currently, standard of care in CKD management includes renin-angiotensin system blockade such as angiotensin-converting enzyme inhibitors and angiotensin II receptor blockers to target glomerular hemodynamics and decrease proteinuria, and consideration of sodium-glucose cotransporter-2 inhibitors. No approved therapies currently target control of uremic toxins, which may have a role in CKD progression. (Lau et al., 1979; Solerte et al., 1987; Kidney Disease Outcomes Quality Initiative, 2004).

With the presence of gut dysbiosis accompanied by reduced renal elimination, gut-derived uremic toxins such as indoxyl sulfate and p-cresyl sulfate are elevated in CKD patients and promote a vicious cycle of kidney damage and disease progression. (Schulman et al., 2014; Asai et al., 2019; Rysz et al., 2021). In 1991, Japan approved AST-120 (Kremezin®), an oral spherical activated carbon with a diameter of approximately 0.2–0.4 mm, for the treatment of CKD. The drug can adsorb acidic and basic organic compounds, especially small-molecule uremic toxins. The main effects of the drug are delaying the need for kidney dialysis and improving the symptoms of uremia. (Asai et al., 2019). However, a previous systematic review indicated that evidence from randomized controlled trials (RCTs) did not conclusively support the benefits of AST-120 in delaying the progression of CKD. Although AST-120 can relieve uremic symptoms and data from large studies suggest that AST-120 can prevent the deterioration of renal function in patients with CKD, the results from other RCTs remain inconclusive. (Asai et al., 2019).

The most recent meta-analysis on this topic was published in 2019, and it reported high heterogeneity (I^2^ = 65%, *p* = 0.04) concerning changes in serum indoxyl sulfate levels. (Chen et al., 2019). It is known that the drug administration strategy is a common cause of heterogeneity (Gagnier et al., 2012; Higgins et al., 2019), and thus, the pooled results of previous meta-analyses may be associated with the treatment strategies of AST-120. Unfortunately, there has been no further discussion concerning the relationship between heterogeneity and treatment strategies. Because multiple treatment strategies were used for AST-120 in previous RCTs, network meta-analysis is an appropriate analysis method because the consistency model compares multiple treatments by combining direct and indirect evidence to assess the effects of each intervention. (Higgins et al., 2019). Therefore this systematic review with network meta-analysis evaluated the effects of AST-120 in patients with CKD, consequently providing integrated evidence-based information for clinical practice.

## Materials and Methods

This study was conducted according to the Cochrane Handbook, and the review team consisted of clinical pharmacists, a nephrologist, and an experienced researcher. The study process and findings were reported according to PRISMA guidance. (Hutton et al., 2015). Because the findings were based on published data, institutional review board approval was not necessary. The synthesis protocol was registered before the comprehensive search.

### Eligibility Criteria and Evidence Selection

According to the purpose of this synthesis, evidence selection was based on eligibility criteria as follows: 1) the study recruited patients with CKD, 2) the intervention was AST-120, and 3) the study was an RCT. However, some references could not be included in the present synthesis because they were inaccessible or gray literature without details. These criteria were defined before the start of the comprehensive search.

The literature search was performed using the Cochrane Database of Systematic Reviews (including Cochrane CENTRAL), Embase, PubMed, and Web of Science using the keywords CKD, AST-120, and related terms. The search was performed using free text, medical subject headings, and abbreviations. The keywords were combined using the Boolean operator “OR,” and the two concepts were connected by the Boolean operator “AND.” No filter was applied for article type, language, and publication date. The primary search strategy was developed in PubMed and adopted to the other databases. The latest search was done by February 5, 2021. The search details are presented in Supplementary File 1.

Two authors used EndNote X9 for the evidence selection process. Duplicates were excluded when the references were imported into EndNote, and the authors manually removed the remaining duplicates. They individually screened and checked references during title and abstract screening and full-text review according to the eligibility criteria. The experienced researcher participated in the evidence selection when the two authors failed to reach a consensus regarding study eligibility, and a final decision was reached after discussion.

### Data Extraction and Quality Evaluation

The same two authors used Microsoft Word to record relevant information and Microsoft Excel to record outcome data. They respectively identified information about trial design, inclusion year, location, CKD stage, treatments, mean age, sex, baseline serum creatinine, and eGFR. They independently extracted and checked outcome data, including mortality, end-stage renal disease, the creatinine clearance rate, the serum creatinine slope, changes in serum indoxyl sulfate and urinary protein levels, and composite renal outcomes. The composite renal outcomes were clinical outcomes related to disease progression, such as dialysis initiation, kidney transplantation, doubling of serum creatinine levels, provided by the trials included. In addition to mortality, end-stage renal disease, and composite renal outcomes, other outcomes were continuous measurements. Thus, we mainly extracted the mean and standard deviation (SD) for the creatinine clearance rate, serum creatinine slope, and changes in serum indoxyl sulfate and urinary protein levels. If the original studies reports did not report data as the mean or SD, the median, standard error (SE), or interquartile range (IQR) were extracted for those outcomes. SDs were estimated from SEs according to the formula SE = SD/√N and from IQR based on the formula IQR/1.35. (Hozo et al., 2005).

Based on the identified information for study design and outcome, two authors evaluated the risk of bias in each RCT using the Cochrane Risk of Bias Tool. They evaluated randomization and concealment for selection bias, blinding of patients and researchers for performance bias, blinding of assessors and follow-up duration for bias detection, and loss to follow-up and type of analysis for attrition bias. The experienced researcher participated in the quality evaluation to resolve disagreements between the two authors.

### Evidence Synthesis and Statistical Analysis

The present study included both qualitative and quantitative analyses. The qualitative synthesis examined the characteristics and patterns of the eligible RCTs. Quantitative analysis was performed using frequentist network meta-analysis. The main outcomes were mortality, end-stage renal disease, and composite renal outcomes. Because the main outcomes were all dichotomous measurements, the consistency model was based on the number of events and sample size in each group, and risk ratios (RRs) were calculated with 95% confidence intervals (CIs). For continuous measurements, the weighted mean difference (WMD) and 95% CI were presented. The surface under the cumulative ranking was further determined to provide more information to clinicians, and this statistical technique estimated the probability of the most effective treatment in each study arm. The study arm ranking of probability was performed using a hierarchy of the best choice of the AST-120 treatment strategy. The quality of consistency model was evaluated by detecting inconsistency and small-study effects. Inconsistency was tested using the Lu–Ades method because some loops existed in the present consistency model, and loop inconsistency should be evaluated. Adjusted funnel plots and Egger’s regression intercept were calculated to evaluate small-study effects. As a common rule, *p* < 0.05 served as the threshold for statistical significance. All analyses in this study were performed using STATA version 14 for Microsoft Windows, and outcomes with complete network were mainly carried out based on a confidence in network meta-analysis platform. The quality of network meta-analysis was further evaluated according to within-study bias, reporting bias, indirectness, imprecision, heterogeneity, and incoherence. (Nikolakopoulou et al., 2020).

## Results

**FIGURE 1:**
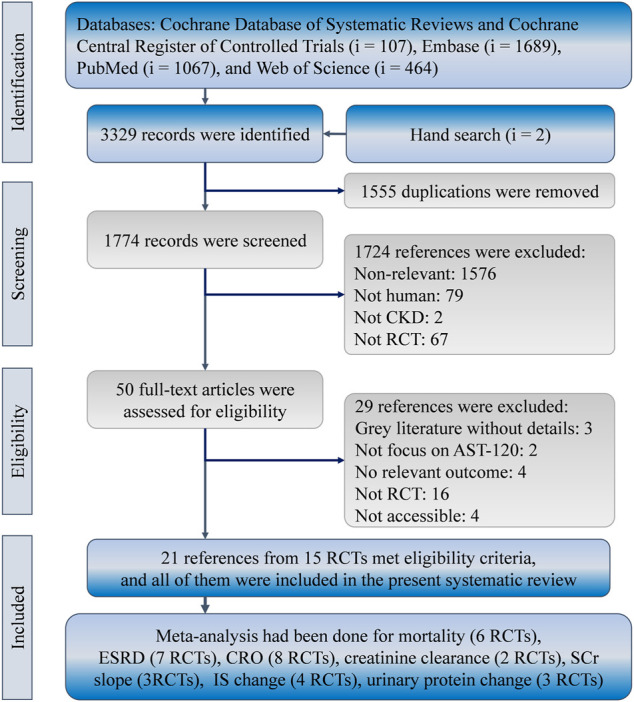
Flowchart of the systematic review and meta-analysis according to PRISMA guidelines. CKD, chronic kidney disease; CRO, composite renal outcome; IS, indoxyl sulfate; RCT, randomized controlled trial; SCr, serum creatinine.

The present study identified 3,327 references in the Cochrane Database of Systematic Reviews (*i* = 107), Embase (*i* = 1,689), PubMed (*i* = 1,067), and Web of Science (*i* = 464). Two more records were found from hand searches of the reference lists. In total, 1,555 duplicates were removed using the “Find duplicates” function in EndNote and manual screening. Afterward, 1774 references were reviewed for eligibility, and 1724 references were eliminated because of a lack of relevance (*i* = 1,576), the inclusion of non-human cohorts (*i* = 79), the inclusion of non-CKD populations (*i* = 2), and a study type other than RCT (*i* = 67). Hence, 50 studies were subjected to full-text review, and 29 of them were not eligible because they were gray literature without details (*i* = 3), they did not report relevant outcomes (*i* = 4), they did not focus on AST-120 (*i* = 2), they were not RCTs (*i* = 16), or they were inaccessible (*i* = 4). Finally, 21 references for 15 RCTs met the eligibility criteria of the present study (Figure 1). (Sanaka et al., 1988; Koide et al., 1991; Owada et al., 1997; Nakamura et al., 2004; Morita et al., 2005; Marier et al., 2006; Schulman et al., 2006; Shoji et al., 2007; Asano et al., 2008; Konishi et al., 2008; Takahashi et al., 2008; Yorioka et al., 2008; Akizawa et al., 2010; Toyoda et al., 2014; Wu et al., 2014; Schulman et al., 2015; Cha et al., 2016; Schulman et al., 2016; Baek et al., 2017; Cha et al., 2017; Schulman et al., 2018)

### Characteristics and Quality of Included Studies

**FIGURE 2:**
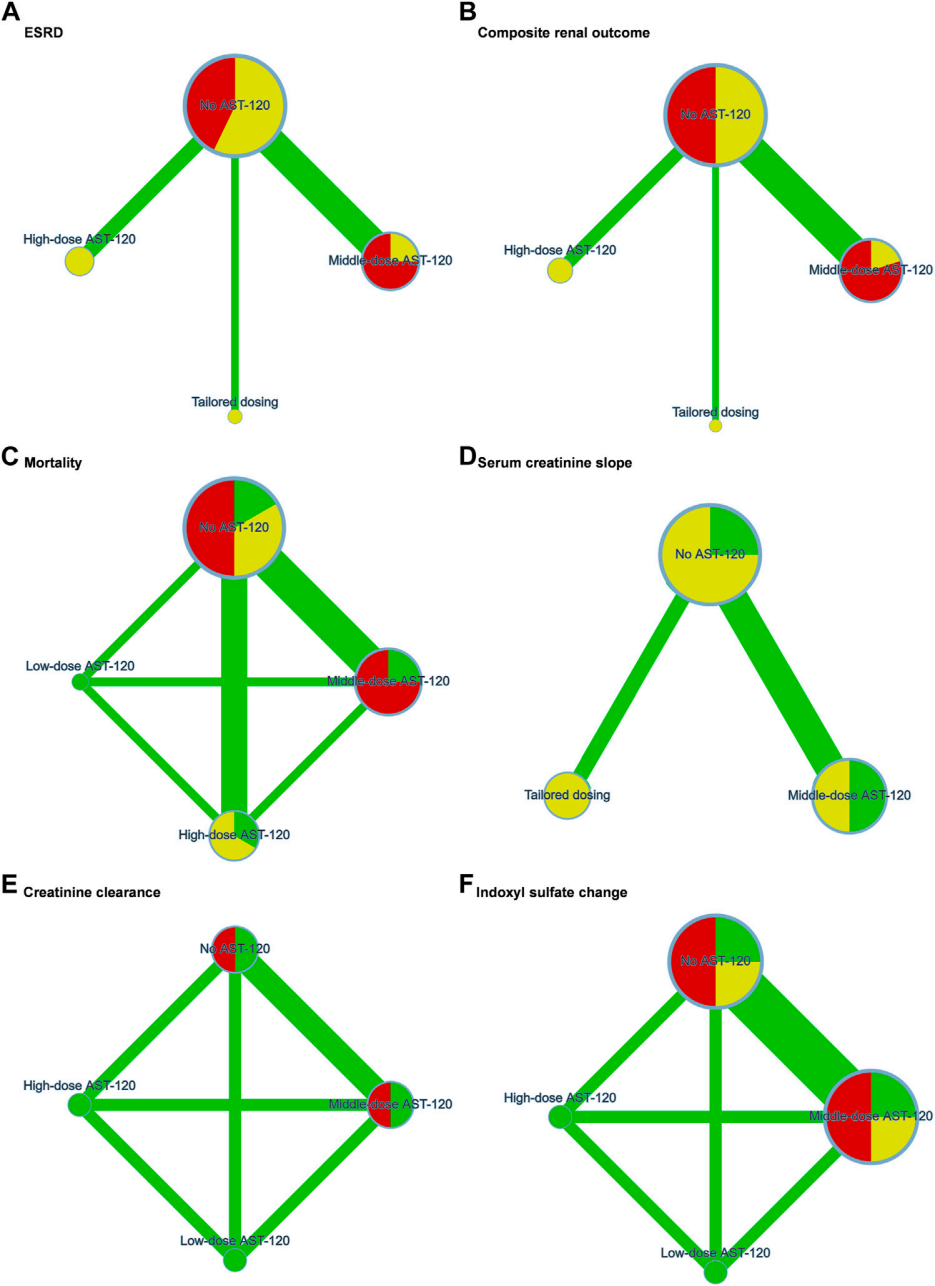
Network plots of **(A)** end stage renal disease, **(B)** composite renal outcome, **(C)** mortality, **(D)** serum creatinine slope, **(E)** creatinine clearance, and **(F)** indoxyl sulfate change. ESRD, end stage renal disease. Green, yellow, and red refers to low risk, some concerns, and high risk of bias respectively.

The 15 trials recruited 3,763 patients undergoing dialysis mainly from Japan, Korea, Taiwan, and the United States. The interventions in the 15 trials could be classified as follows: no AST-120, low-dose AST-120 (approximately 3 g), middle-dose AST-12 (approximately 6 g), high-dose AST-120 (9 g), and tailored dosing. Three studies used tailored dosing, while protocols of tailored dosing varied by trials. Tailored dosing among them ranged from 3 g/d to 7.2 g/d. Tailored AST-120 treatment strategies in most of the trials were initiated with lower dose (3 g/d or 3.2 g/d), and gradually increased to middle-dose. The consistency model of each primary outcome is presented in Figure 2. Based on the available data in each study, the mean age of the recruited patients ranged from 44.7 to 71.6 years old, and a total of 2020 (53.68%) patients were male. Table 1 presents the trial location, inclusion years, intervention strategy, and dialysis period. The quality of the RCTs is highlighted in Supplementary File 2.

**TABLE 1:** Characteristics of the included randomized controlled trials.

Author	Location	Inclusion	Main treatments		Mean	Sex
		Year	Dose	Duration	age	(M/F)
Sanaka et al. (1988)	Japan	NR	I: Tailored AST-120 (3.2 g/d-7.2 g/d)	4.2–12.6 months	44.7	19/8
			C: Usual care		46.8	15/9
Koide et al. (1991)	Japan	1982–1983	Study 1:	6 years	NR	Overall: 566
			I: tailored AST-120 (initial 3 g/d → 5–6 g/d)			
			Study 2:	24 weeks	NR	Overall: 244
			I: AST-120 6 g/d			
			C: Usual care			
Owada et al. (1997)	Japan	NR	I: tailored AST-120 (initial 3 g/d → 6 g/d)	12–24 months	NR	Overall: 13
			C: Usual care			
Nakamura et al. (2004)	Japan	NR	I: AST-120 6 g/d	24 months	53.5	18/12
			C: Usual care		52	12/8
Marier et al. (2006)	Japan	NR	I: AST-120 9 g/d	7 days	Overall: 58	Overall: 10
			C: Usual care			
Schulman et al. (2006)	United States	NR	I: AST-120 9 g/day	3 months	69.3	30/9
			AST-120 6.3 g/day		66.3	22/18
			AST-120 2.7 g/day		59.6	30/9
			C: Usual care		63.1	25/14
Shoji et al. (2007)	Japan	NR	I: AST-120 6 g/d	12 months	54.1	11/3
			C: Usual care		54.5	10/2
Konishi et al. (2008)	Japan	2001	I: AST-120 6 g/d	37 months	52	2/4
			C: Usual care		56	7/3
Yorioka et al. (2008)	Japan	2003–2006	I: AST-120 6 g/day	12 months	61.7	11/4
			C: Usual care		59.7	5/8
Akizawa et al. (2009) (CAP-KD)	Japan	NR	I: AST-120 6 g/d	56 weeks	62.9	80/151
			C: Usual care		63.3	73/156
Toyoda et al. (2014)	Japan	2012	I: AST-120 6 g/day	24 weeks	71.5	42/13
			C: Usual care		71.6	45/20
Wu et al. (2014)	Taiwan	NR	I: AST-120 6 g/day	12 weeks	Overall	Overall
			C: Usual care		61.26	16/35
Schulman et al. (2015) (EPIC-1)	Multiple countries	2007–2012	I: AST-120 9 g/day	102.1 weeks	56.3	309/191
			C: Usual care	103.3 weeks	55.6	326/176
Schulman et al. (2015) (EPIC-2)	Multiple countries	2007–2012	I: AST-120 9 g/day	96.3 weeks	54.4	273/227
			C: Usual care	91.6 weeks	55.5	276/221
Cha et al. (2016) (K-star)	Korea	2009–2010	I: AST-120 6 g/d	12 months	56.7	185/87
			C: Usual care	24 months	56.8	178/88
				36 months		

C, control group; I, intervention group; M/F, male/female; NR, no report.

### Renal Events

**FIGURE 3:**
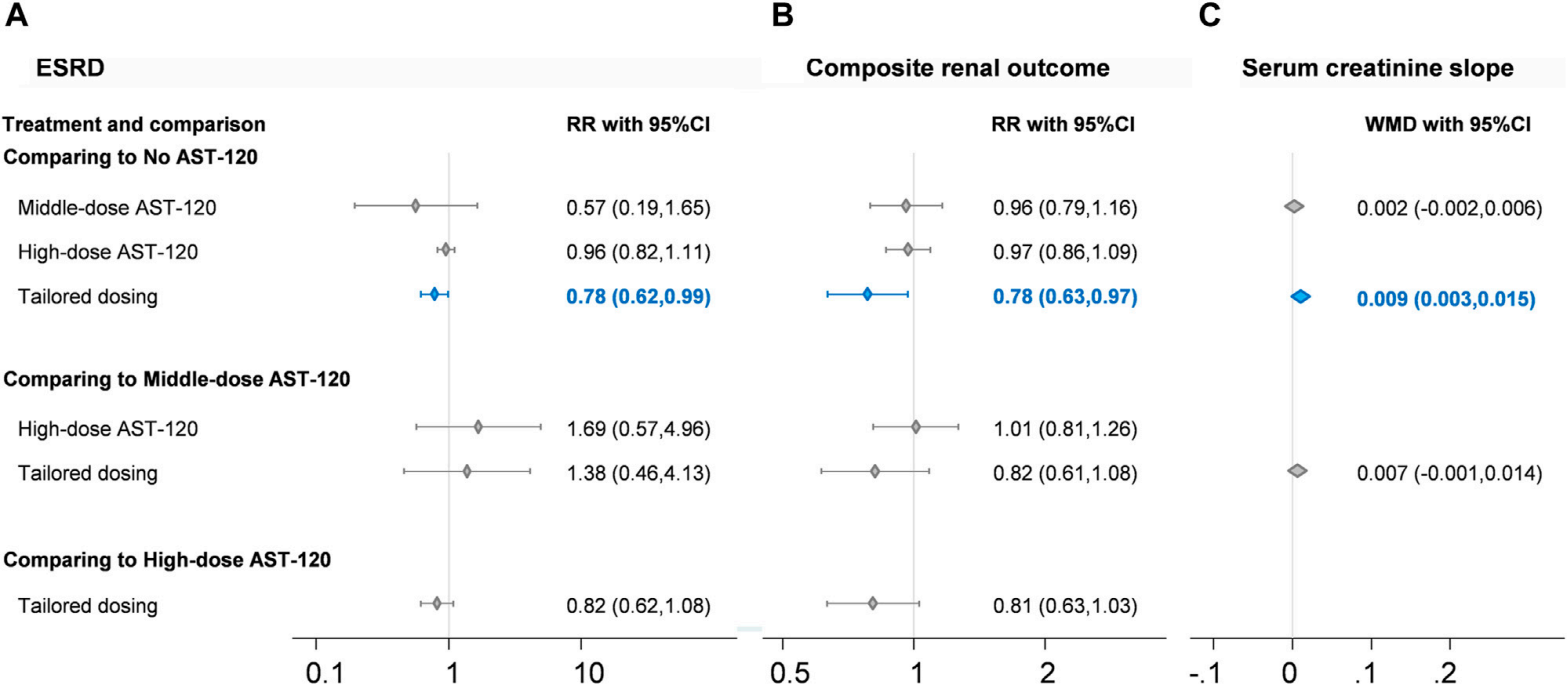
Forest plots of **(A)** end stage renal disease, **(B)** composite renal outcome, and **(C)** serum creatinine slope. CI, confidence interval; ESRD, end stage renal disease; RR, risk ratio. Blue diamond and line indicate statistically significant findings.

Concerning renal events, data were available for end-stage renal disease event and composite renal outcomes. Seven RCTs reported data on end-stage renal disease events (Sanaka et al., 1988; Konishi et al., 2008; Yorioka et al., 2008; Akizawa et al., 2010; Wu et al., 2014; Schulman et al., 2015; Schulman et al., 2016; Schulman et al., 2018), and 2,658 patients in the RCTs were included in the corresponding network meta-analysis. Four treatment strategies formed the network meta-analysis, including no AST-120, middle-dose AST-120, high-dose AST-120, and tailored dosing. No statistically significant difference in end-stage renal disease event rates was identified among the no AST-120, middle-dose AST-120, and high-dose AST-120 groups (Figure 3A), whereas the event rate was significantly lower in the tailored dosing group than in no AST-120 group (RR = 0.78; 95% CI: 0.62–0.99). The heterogeneity of each direct comparison was low (Supplementary File 3), yet inconsistency testing could not be performed because of the paucity of loops in the consistency model. A small-study effect was not detected (coefficient = −0.13; *p* > 0.1; Supplementary File 4).

Concerning composite renal outcomes, eight RCTs involving 3,196 patients were included in the four-node network meta-analysis of mortality (Sanaka et al., 1988; Schulman et al., 2006; Konishi et al., 2008; Yorioka et al., 2008; Akizawa et al., 2010; Wu et al., 2014; Schulman et al., 2015; Cha et al., 2016; Schulman et al., 2016; Cha et al., 2017; Schulman et al., 2018), and the four-node network also consisted of no AST-120, middle-dose AST-120, high-dose AST-120, and tailored dosing groups. The composite renal outcome event rate was significantly lower in the tailored dosing group than in no AST-120 group (RR = 0.78; 95% CI: 0.63–0.97; Figure 3B), and no other comparisons in the consistency model revealed significant differences in the rates of composite renal outcomes. This finding was similar to the result for end-stage renal disease. Because the consistency model of composite renal outcomes had no loops, no inconsistency test could be conducted, but the heterogeneity of each direct comparison was low (Supplementary File 5). Moreover, no evidence of serious small-study effects was identified (coefficient = −0.49; *p* > 0.1; Supplementary File 6).

### Mortality

A total of six RCTs including 3,250 patients were included in the four-node network meta-analysis of mortality. (Schulman et al., 2006; Akizawa et al., 2010; Wu et al., 2014; Schulman et al., 2015; Cha et al., 2016; Schulman et al., 2016; Cha et al., 2017; Schulman et al., 2018). The results revealed no significant finding in the consistency model, although the large effect size, albeit without statistical significance, between the high-dose AST-120 and no AST-120 groups might raise clinical concerns (RR = 0.48; 95% CI: 0.22–1.05). Our analysis did not detect evident loop inconsistency (chi-square = 0.10; *p* > 0.1), but a small-study effect was noted (coefficient = −0.26; *p* > 0.1).

### Secondary Findings

Some laboratory data were presented in the included RCTs; and four-node network meta-analysis of no AST-120, low-dose AST-120, middle-dose AST-120, and high-dose AST-120 could be built for creatinine clearance rates, serum creatinine slope, changes in indoxyl sulfate levels, and changes in urinary protein levels. The serum creatinine slope was apparently improved after tailored dosing compared to the effects of no AST-120 (WMD = 0.009; 95% CI: 0.003 to 0.015; Figure 3C), and changes in indoxyl sulfate levels were apparently lower in middle-dose AST-120 compared to the effects of no AST-120 (WMD = −0.517; 95% CI: −1.013 to −0.020; Table 2). However, consistency models did not reveal any significant differences in creatinine clearance rates and changes in urinary protein change, although the trends appeared to favor middle-dose AST-120 concerning urinary protein levels.

**TABLE 2:** League tables.

Comparator outcome	No AST-120	Low-dose AST-120	Middle-dose AST-120
Mortality^a^			
Low-dose AST-120	0.692 (0.024, 20.208)		
Middle-dose AST-120	0.833 (0.274, 2.532)	1.203 (0.039, 37.189)	
High-dose AST-120	0.485 (0.200, 1.175)	0.701 (0.023, 21.187)	0.582 (0.144, 2.348)
Creatinine clearance^b^			
Low-dose AST-120	−1.147 (−4.526, 2.232)		
Middle-dose AST-120	−0.555 (−2.425, 1.314)	0.592 (−2.743, 3.926)	
High-dose AST-120	1.563 (−2.753, 5.879)	2.710 (−1.942, 7.362)	2.118 (−2.163, 6.400)
Indoxyl sulfate change^b^			
Low-dose AST-120	−0.181 (−0.862, 0.500)		
Middle-dose AST-120	−0.517 (−1.013, -0.020)	−0.336 (−1.016, 0.344)	
High-dose AST-120	−0.491 (−1.169, 0.188)	−0.310 (−1.039, 0.419)	0.026 (−0.651, 0.703)
Urinary protein change^b^			
Low-dose AST-120	0.130 (−0.472, 0.732)		
Middle-dose AST-120	−0.132 (−0.343, 0.078)	−0.262 (−0.852, 0.327)	
High-dose AST-120	0.100 (−0.444, 0.644)	−0.030 (−0.718, 0.657)	0.232 (−0.298, 0.762)

a Risk ratio (95% confidence interval).

b Mean difference (95% confidence interval).

## Discussion

### Key Findings

We pooled evidence from RCTs that compared different doses and treatment strategies to simultaneously estimate the relative effectiveness of AST-120 against kidney disease outcomes in individuals with CKD stages 3–5 who did not require dialysis, thereby overcoming the lack of comparative data in head-to-head trials. The present synthesis updated this topic using 15 RCTs involving 3,763 patients, and the pooled results demonstrated the potential benefits of AST-120 in patients with CKD based on its ability to reduce composite renal outcomes, prevent the progression to end-stage renal disease rate, correct serum creatinine slope, and indoxyl sulfate level. Remarkably, middle-dose or tailored treatment with AST-120 had statistically significant benefits.

Although AST-120 did not appear to have significant effects on renal function markers in the acute phase (Marier et al., 2006), the present synthesis identified the effects of AST-120 on clinical outcomes. Before our study, one meta-analysis of 1,590 patients investigated the benefits and drawbacks of oral adsorbents for preventing or delaying the progression of CKD, but the article failed to report clinical relevant outcomes, such as the incidence of and time to end-stage renal disease and all-cause mortality, from 15 studies published between 1976 and 2012 to support the clinical use of AST-120. (Wu et al., 2014). Another review in 2019 only provided an overview of 10 prior clinical studies on AST-120 published from 1982 to 2013. (Asai et al., 2019). We know that some methodological barriers may have limited their confidence in recommending AST-120 for patients with CKD, but we agree with their conclusions and concerns. Because the definition of CKD progression and outcome measurements in some post hoc analyses differed from those in other recent RCTs, the overall clinical benefit of AST-120 in CKD management should be verified by additional studies based on objective data, particularly laboratory data. Although the present synthesis reported pooled estimates of creatinine clearance rates, serum creatinine slopes, and change in indoxyl sulfate and urinary protein levels, the pooled findings may be not robust because of the small sample size (Table 3). These laboratory findings are important to clinicians in understanding the effects of AST-120 on the kidneys.

**TABLE 3:** Confidence rating for findings from complete network meta-analysis.

Comparison	Studies	Within-study bias	Reporting bias	Indirectness	Imprecision	Heterogeneity	Incoherence	Confidence rating
Mortality								
HD vs LD	1	No concerns	Undetected	No concerns	Major concerns	No concerns	No concerns	Moderate
HD vs MD	1	Some concerns	Undetected	No concerns	Major concerns	No concerns	No concerns	Low
HD vs Non	3	Some concerns	Undetected	No concerns	Some concerns	Some concerns	No concerns	Moderate
LD vs MD	1	Some concerns	Undetected	No concerns	Major concerns	No concerns	No concerns	Low
LD vs Non	1	No concerns	Undetected	No concerns	Major concerns	No concerns	No concerns	Low
MD vs Non	4	Major concerns	Undetected	No concerns	Major concerns	No concerns	No concerns	Very low
Creatinine clearance								
HD vs Non	1	No concerns	Undetected	No concerns	No concerns	No concerns	No concerns	High
MD vs Non	1	No concerns	Suspected	No concerns	No concerns	No concerns	No concerns	Low
Non vs TD	1	No concerns	Undetected	No concerns	No concerns	No concerns	No concerns	High
HD vs MD	1	No concerns	Undetected	No concerns	No concerns	No concerns	No concerns	High
HD vs TD	1	No concerns	Undetected	No concerns	No concerns	No concerns	No concerns	High
MD vs TD	2	Some concerns	Undetected	No concerns	No concerns	No concerns	No concerns	Moderate
Indoxyl sulfate change								
HD vs LD	1	No concerns	Undetected	No concerns	No concerns	No concerns	No concerns	High
HD vs MD	1	No concerns	Undetected	No concerns	No concerns	No concerns	No concerns	High
HD vs Non	1	No concerns	Undetected	No concerns	No concerns	No concerns	No concerns	High
LD vs MD	1	No concerns	Undetected	No concerns	No concerns	No concerns	No concerns	High
LD vs Non	1	No concerns	Undetected	No concerns	No concerns	No concerns	No concerns	High
MD vs Non	4	No concerns	Undetected	No concerns	No concerns	No concerns	No concerns	High
Urinary protein change								
HD vs LD	1	No concerns	Undetected	No concerns	No concerns	Some concerns	No concerns	Moderate
HD vs MD	1	No concerns	Suspected	No concerns	No concerns	Some concerns	No concerns	Low
HD vs Non	1	No concerns	Undetected	No concerns	No concerns	Some concerns	No concerns	Moderate
LD vs MD	1	No concerns	Undetected	No concerns	No concerns	Some concerns	No concerns	Moderate
LD vs Non	1	No concerns	Undetected	No concerns	No concerns	Some concerns	No concerns	Moderate
MD vs Non	3	Major concerns	Undetected	No concerns	No concerns	Major concerns	No concerns	Very low

HD, high-dose AST-120; LD, low-dose AST-120; MD, middle-dose AST-120; Non, no AST-120; TD, tailored dosing AST-120.

Regarding the potential molecular mechanism, AST-120 is hypothesized to improved kidney function by decreasing the levels of nephrotoxic metabolites in serum, such as advanced glycation end products, indoxyl sulfate, and p-cresol. (Mosińska et al., 2015). Of these, indoxyl sulfate has been recognized to be associated with kidney disease progression. In the present synthesis, the changes of serum indoxyl sulfate levels were smaller in patients treated with AST-120 than in those who did not receive the drug, but no significant difference was found in each pairwise comparison. Nevertheless, the serum creatinine slope was significantly lessened in patients treated with tailored-dose AST-120 compared to the findings in patients who did not receive AST-120. On the basis of the current work, AST-120 could also steadily decrease the levels of all nephrotoxic metabolites in serum, and its ability to suppress the levels of one uremic toxin could have beneficial effects on kidney progression.

Some trials did not report relevant data for this network meta-analysis, but they still support the renal protective effects of AST-120. For instance, an early trial in Japan indicated that AST-120 could delay the time to dialysis (Koide et al., 1991), but the trial did not have available data for our pooled analysis. Another post hoc study also revealed a difference between AST-120 and placebo concerning the time to the composite endpoint (e.g., initiation of dialysis or doubling of serum creatinine levels), although no data were available for the present synthesis. (Asai et al., 2019). In other words, evidence appeared to be consistent, but, the effects of AST-120 may differ among the treatment strategies.

### Optimal Medication Strategy of AST-120

In fact, we noted that tailored treatment with AST-120 or moderate dosing (6 g per day) may represent the optimal strategy. Adherence might be an important factor supporting the efficacy of AST-120 in clinical practice. A post hoc analysis using a per-protocol strategy revealed that patients who received 6 g per day had higher compliance and lower composite event rates than those receiving standard care. (Cha et al., 2017). Patients in both the AST-120 and standard care groups had similar characteristics at baseline. Hence, the association between dose and adherence may not be seriously biased by the initial conditions.

Adverse events appear to explain, at least in part, the influence of adherence on the effects of AST-120 on renal outcomes. One recent meta-analysis indicated that patients treated with tailored-dose AST-120 (dose increases from 2.7 to 9 g per day) experienced more dermatological events than those treated with placebo or conventional care, and thus, the incidence of adverse events was higher in the tailored dosing than in the control group. (Schulman et al., 2006; Laufs et al., 2010; Schulman et al., 2015; Chen et al., 2019). Patient adherence is usually decreased by increased rates of adverse events. (Hsu et al., 2015). Consequently, dose-related adverse events may be one reason why high-dose AST-120 was less effective than tailored-dose or middle-dose AST-120.

Tailored treatment with AST-120, in which each patient was treated at a dose of 3.2–7.2 g per day for 2.4–30.1 months, had suppressive effects on the progression of chronic renal failure, such as blocking the elevation of serum creatinine levels, improving uremic symptoms, and therefore delaying the initiation of dialysis therapy. (Sanaka et al., 1988). Similar results were also reported in another trial that using tailored dose of AST-120 from 3 g/d to 6 g/d (Owada et al., 1997). The benefits of tailored treatment in which patients were started at an AST-120 dose of 3 g per day followed by subsequent increases to reach a maintenance dose of 5–6 g per day were also indicated in early trials, including prolongation of the period of hemodialysis. (Koide et al., 1991).

### Limitations

Our study had several limitations. We examined studies published between 1980 and 2012, and the classification of kidney function changed over this period. In addition, subgroup analysis could not be conducted because of the lack of data. Fortunately, most outcomes in the present study were not affected by the different classifications of kidney function, such as mortality, dialysis events, and laboratory data. Second, tailored AST-120 varied among the included trials, and the clinical heterogeneity may weaken the evidence certainty. However, we have noticed that the various practice of tailored AST-120 did not strongly affect the results since the studies using different protocol for individualized medication of AST-120 had a similar trend favoring tailored treatment. Third, our synthesis revealed some trends regarding the benefits of AST-120 in patients with CKD according to laboratory data, but the results may be underpowered because of the small sample size. Our primary outcomes were based on more evidence, and they exhibited similar trends as the laboratory data. More laboratory data are expected to be reported in the future, and these data should improve our understanding of the influence of AST-120 on kidney function.

## Conclusion

This is the first network meta-analysis on AST-120 (Kremezin) in patients with CKD. AST-120, particularly tailored treatment or middle-dose therapy (approximately 6 g per day), appears to reduce composite renal outcomes and progression to end-stage renal disease. High-dose (9 g per day) or low-dose therapy (approximately 3 g per day) are probably not appropriate for this population. Thus, titration from the initial dose to the maintenance dose according to patient adherence and adverse effects is recommended.

## Data Availability

The original contributions presented in the study are included in the article/Supplementary Material, further inquiries can be directed to the corresponding authors.

## Abbreviations

CI, confidence interval; CKD, chronic kidney disease; IQR, interquartile range; PRISMA, preferred reporting items for systematic reviews and meta-analyses; RCT, randomized controlled trial; RR, risk ratio; SD, standard deviation; SE, standard error; UI, uncertainty interval; WMD, weighted mean difference.
