# Supplementary material for: Efficacy of AST-120 for Patients With Chronic Kidney Disease: A Network Meta-Analysis of Randomized Controlled Trials
Source: Front Pharmacol. 2021 Jul 26;12:676345. doi: 10.3389/fphar.2021.676345 (PMC8350440; doi:10.3389/fphar.2021.676345)

Frontiers in Pharmacology

Efficacy of AST-120 for Patients with Chronic Kidney Disease:

A Network Meta-analysis of Randomized Controlled Trials

(Supplementary File)

Authors:

Pei-Yu, Su RPh., M.S.[1,+], **Ya-Han, Lee** RPh., M.S.[1,2,+], **Li-Na Kuo,** RPh., M.S.[1,2], **Yen-Cheng Chen** M.D.[3], **Chiehfeng Chen** M.D., Ph.D., Prof.[4,5,6,7], **Yi-No Kang** M.A.[4,5,8,9,*], **Elizabeth H. Chang**, Pharm.D., Ph.D.[1,2,10]

Affiliations:

1. Department of Pharmacy, Wan Fang Hospital, Taipei Medical University, Taipei, Taiwan
2. Department of Clinical Pharmacy, School of Pharmacy, Taipei Medical University, Taipei, Taiwan
3. Division of Nephrology, Department of Internal Medicine, Wan Fang Hospital, Taipei Medical University, Taiwan
4. Cochrane Taiwan, Taipei Medical University, Taipei, Taiwan
5. Evidence-Based Medicine Center, Wan Fang Hospital, Taipei Medical University, Taipei, Taiwan
6. Division of Plastic Surgery, Department of Surgery, Wan Fang Hospital, Taipei Medical University, Taipei, Taiwan
7. Department of Public Health, School of Medicine, College of Medicine, Taipei Medical University, Taipei, Taiwan
8. Research Center of Big Data and Meta-analysis, Wan Fang Hospital, Taipei Medical University, Taipei, Taiwan
9. Institute of Health Policy and Management, College of Public Health, National Taiwan University, Taipei, Taiwan
10. Research Center for Pharmacoeconomics, College of Pharmacy, Taipei Medical University, Taipei, Taiwan

Index of Supplementary File

Supplementary File 1. Search strategy

Supplementary File 2. Quality assessment (CINeMA approach)

Supplementary File 3. Forest plot of direct evidence on end stage renal disease

Supplementary File 4. Publication bias in network meta-analysis of end stage renal disease

Supplementary File 5. Forest plot of direct evidence on composite renal outcome

Supplementary File 6. Publication bias in network meta-analysis of composite renal outcome

Supplementary File 1
Search strategy

Supplementary File 1
Search strategy

Primary search strategy
#1 renal replacement therapy
#2 dialysis
#3 hemodialysis
#4 haemodialysis
#5 Renal Insufficiency
#6 Kidney Failure, Chronic
#7 chronic kidney failure
#8 chronic kidney insufficiency
#9 chronic renal failure
#10 chronic renal insufficiency
#11 kidney chronic failure
#12 kidney failure, chronic
#13 renal insufficiency, chronic
#14 CKD
#15 ESRD
#16 end stage renal disease
#17 ESKD
#18 end stage kidney disease
#19 #1 OR #2 OR #3 OR #4 OR #5 OR #6 OR #7 OR #8 OR #9 OR #10 OR #11 OR #12 OR #13 OR #14 OR #15 OR #16 OR #17 OR #18
#20 Kremezin
#21 Merckmezin
#22 Kyucal
#23 spherical absorptive carbon
#24 spherical absorptive carbons
#25 adsorbent carbons
#26 adsorbent carbon
#27 sorbent
#28 sorbents
#29 adsorbent
#30 adsorbents
#31 AST-120
#32 #20 OR #21 OR #22 OR #23 OR #24 OR #25 OR #26 OR #27 OR #28 OR #29 OR #30 OR #31
#33 #31 AND #32

Embase
('renal support'/exp OR 'renal support' OR 'chronic kidney disease'/exp OR 'chronic kidney disease' OR 'chronic kidney disorder'/exp OR 'chronic kidney disorder' OR 'chronic kidney failure'/exp OR 'chronic kidney failure' OR 'chronic kidney insufficiency'/exp OR 'chronic kidney insufficiency' OR 'chronic nephropathy'/exp OR 'chronic nephropathy' OR 'chronic renal disease'/exp OR 'chronic renal disease' OR 'chronic renal failure'/exp OR 'chronic renal failure' OR 'chronic renal insufficiency'/exp OR 'chronic renal insufficiency' OR 'kidney chronic failure'/exp OR 'kidney chronic failure' OR 'kidney disease, chronic'/exp OR 'kidney disease, chronic' OR 'kidney failure, chronic'/exp OR 'kidney failure, chronic' OR 'kidney function, chronic disease'/exp OR 'kidney function, chronic disease' OR 'renal insufficiency, chronic'/exp OR 'renal insufficiency, chronic' OR ckd OR 'esrd'/exp OR 'esrd' OR 'end stage kidney disease'/exp OR 'end stage kidney disease' OR 'end stage kidney failure'/exp OR 'end stage kidney failure' OR 'end stage renal disease'/exp OR 'end stage renal disease' OR 'end stage renal dysfunction'/exp OR 'end stage renal dysfunction' OR 'end stage renal failure'/exp OR 'end stage renal failure' OR 'end stage renal impairment'/exp OR 'end stage renal impairment' OR 'end stage renal insufficiency'/exp OR 'end stage renal insufficiency' OR 'end-stage kidney disease'/exp OR 'end-stage kidney disease' OR 'end-stage kidney failure'/exp OR 'end-stage kidney failure' OR 'end-stage renal disease'/exp OR 'end-stage renal disease' OR 'stage 5 kidney disease'/exp OR 'stage 5 kidney disease' OR 'stage 5 renal disease'/exp OR 'stage 5 renal disease' OR eskd) AND ('ast 120'/exp OR 'ast 120' OR 'ast120'/exp OR 'ast120' OR 'kremezin'/exp OR 'kremezin' OR merckmezin OR kyucal OR 'spherical absorptive carbon' OR 'spherical absorptive carbons' OR 'adsorbent carbons' OR 'adsorbent carbon' OR sorbent OR 'sorbent'/exp OR 'sorbent' OR sorbents OR adsorbent OR 'adsorbent'/exp OR 'adsorbent' OR 'adsorbent agent'/exp OR 'adsorbent agent' OR adsorbents)

Supplementary File 1
Database and search strategy

PubMed

(renal replacement therapy OR dialysis OR hemodialysis OR haemodialysis OR Renal Insufficiency OR Kidney Failure, Chronic OR chronic kidney failure OR chronic kidney insufficiency OR chronic renal failure OR chronic renal insufficiency OR kidney chronic failure OR kidney failure, chronic OR renal insufficiency, chronic OR CKD OR ESRD OR end stage renal disease OR ESKD OR end stage kidney disease) AND ("Kremezin"[tiab] OR "Merckmezin"[tiab] OR "Kyucal"[tiab] OR "spherical absorptive carbon"[tiab] OR "spherical absorptive carbons"[tiab] OR "adsorbent carbons"[tiab] OR "adsorbent carbon"[tiab] OR sorbent[tiab] OR sorbents[tiab] OR adsorbent[tiab] OR adsorbents[tiab] OR AST-120[tiab])

Search details:
("renal replacement therapy"[MeSH Terms] OR ("renal"[All Fields] AND "replacement"[All Fields] AND "therapy"[All Fields]) OR "renal replacement therapy"[All Fields] OR ("dialysance"[All Fields] OR "dialysances"[All Fields] OR "dialysation"[All Fields] OR "dialysator"[All Fields] OR "dialysators"[All Fields] OR "dialyse"[All Fields] OR "dialysed"[All Fields] OR "dialyser"[All Fields] OR "dialysers"[All Fields] OR "dialysing"[All Fields] OR "dialysis solutions"[Pharmacological Action] OR "dialysis solutions"[MeSH Terms] OR ("dialysis"[All Fields] AND "solutions"[All Fields]) OR "dialysis solutions"[All Fields] OR "dialysate"[All Fields] OR "dialysates"[All Fields] OR "dialyzate"[All Fields] OR "dialyzates"[All Fields] OR "dialysis"[MeSH Terms] OR "dialysis"[All Fields] OR "dialyses"[All Fields] OR "dialyzability"[All Fields] OR "dialyzable"[All Fields] OR "dialyzation"[All Fields] OR "dialyze"[All Fields] OR "dialyzed"[All Fields] OR "dialyzer"[All Fields] OR "dialyzer s"[All Fields] OR "dialyzers"[All Fields] OR "dialyzing"[All Fields] OR "renal dialysis"[MeSH Terms] OR ("renal"[All Fields] AND "dialysis"[All Fields]) OR "renal dialysis"[All Fields]) OR ("haemodialysis"[All Fields] OR "renal dialysis"[MeSH Terms] OR ("renal"[All Fields] AND "dialysis"[All Fields]) OR "renal dialysis"[All Fields] OR "hemodialysis"[All Fields]) OR ("haemodialysis"[All Fields] OR "renal dialysis"[MeSH Terms] OR ("renal"[All Fields] AND "dialysis"[All Fields]) OR "renal dialysis"[All Fields] OR "hemodialysis"[All Fields]) OR ("renal insufficiency"[MeSH Terms] OR ("renal"[All Fields] AND "insufficiency"[All Fields]) OR "renal insufficiency"[All Fields]) OR ("kidney failure, chronic"[MeSH Terms] OR ("kidney"[All Fields] AND "failure"[All Fields] AND "chronic"[All Fields]) OR "chronic kidney failure"[All Fields] OR ("kidney"[All Fields] AND "failure"[All Fields] AND "chronic"[All Fields]) OR "kidney failure chronic"[All Fields]) OR ("kidney failure, chronic"[MeSH Terms] OR ("kidney"[All Fields] AND "failure"[All Fields] AND "chronic"[All Fields]) OR "chronic kidney failure"[All Fields] OR ("chronic"[All Fields] AND "kidney"[All Fields] AND "failure"[All Fields])) OR ("renal insufficiency, chronic"[MeSH Terms] OR ("renal"[All Fields] AND "insufficiency"[All Fields] AND "chronic"[All Fields]) OR "chronic renal insufficiency"[All Fields] OR ("chronic"[All Fields] AND "kidney"[All Fields] AND "insufficiency"[All Fields]) OR "chronic kidney insufficiency"[All Fields]) OR ("kidney failure, chronic"[MeSH Terms] OR ("kidney"[All Fields] AND "failure"[All Fields] AND "chronic"[All Fields]) OR "chronic kidney failure"[All Fields] OR ("chronic"[All Fields] AND "renal"[All Fields] AND "failure"[All Fields]) OR "chronic renal failure"[All Fields]) OR ("renal insufficiency, chronic"[MeSH Terms] OR ("renal"[All Fields] AND "insufficiency"[All Fields] AND "chronic"[All Fields]) OR "chronic renal insufficiency"[All Fields] OR ("chronic"[All Fields] AND "renal"[All Fields] AND "insufficiency"[All Fields])) OR (("kidney"[MeSH Terms] OR "kidney"[All Fields] OR "kidneys"[All Fields] OR "kidney s"[All Fields]) AND ("chronic"[All Fields] OR "chronical"[All Fields] OR "chronically"[All Fields] OR "chronicities"[All Fields] OR "chronicity"[All Fields] OR "chronicization"[All Fields] OR "chronics"[All Fields]) AND ("failure"[All Fields] OR "failures"[All Fields])) OR ("kidney failure, chronic"[MeSH Terms] OR ("kidney"[All Fields] AND "failure"[All Fields] AND "chronic"[All Fields]) OR "chronic kidney failure"[All Fields] OR ("kidney"[All Fields] AND "failure"[All Fields] AND "chronic"[All Fields]) OR "kidney failure chronic"[All Fields]) OR ("renal insufficiency, chronic"[MeSH Terms] OR ("renal"[All Fields] AND "insufficiency"[All Fields] AND "chronic"[All Fields]) OR "chronic renal insufficiency"[All Fields] OR ("renal"[All Fields] AND "insufficiency"[All Fields] AND "chronic"[All Fields]) OR "renal insufficiency chronic"[All Fields]) OR "CKD"[All Fields] OR ("kidney failure, chronic"[MeSH Terms] OR ("kidney"[All Fields] AND "failure"[All Fields] AND "chronic"[All Fields]) OR "chronic kidney failure"[All Fields] OR "esrd"[All Fields]) OR ("kidney failure, chronic"[MeSH Terms] OR ("kidney"[All Fields] AND "failure"[All Fields] AND "chronic"[All Fields]) OR "chronic kidney failure"[All Fields] OR ("end"[All Fields] AND "stage"[All Fields] AND "renal"[All Fields] AND "disease"[All Fields]) OR "end stage renal disease"[All Fields]) OR "ESKD"[All Fields] OR ("kidney failure, chronic"[MeSH Terms] OR ("kidney"[All Fields] AND "failure"[All Fields] AND "chronic"[All Fields]) OR "chronic kidney failure"[All Fields] OR ("end"[All Fields] AND "stage"[All Fields] AND "kidney"[All Fields] AND "disease"[All Fields]) OR "end stage kidney disease"[All Fields])) AND ("Kremezin"[Title/Abstract] OR "Merckmezin"[Title/Abstract] OR "sorbent"[Title/Abstract] OR "sorbents"[Title/Abstract] OR "adsorbent"[Title/Abstract] OR "adsorbents"[Title/Abstract] OR "AST-120"[Title/Abstract])

Web of Science

TOPIC: (Renal Insufficiency OR Kidney Failure, Chronic OR chronic kidney failure OR chronic kidney insufficiency OR chronic renal failure OR chronic renal insufficiency OR kidney chronic failure OR kidney failure, chronic OR renal insufficiency, chronic OR CKD OR ESRD OR end stage renal disease OR ESKD OR end stage kidney disease) AND TOPIC: ("Kremezin" OR "Merckmezin" OR "Kyucal" OR spherical absorptive carbon OR spherical absorptive carbons OR adsorbent carbons OR adsorbent carbon OR sorbent OR sorbents OR adsorbent OR adsorbents OR AST-120)

Supplementary File 2

Quality assessment (CINeMA approach)

Supplementary File 2
Quality assessment (CINeMA approach)

Mortality

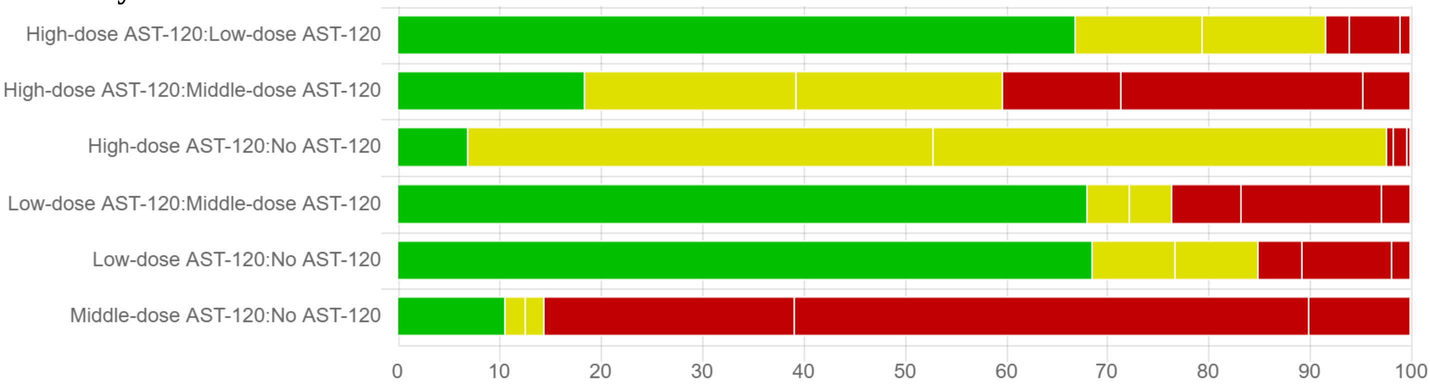

End stage renal disease

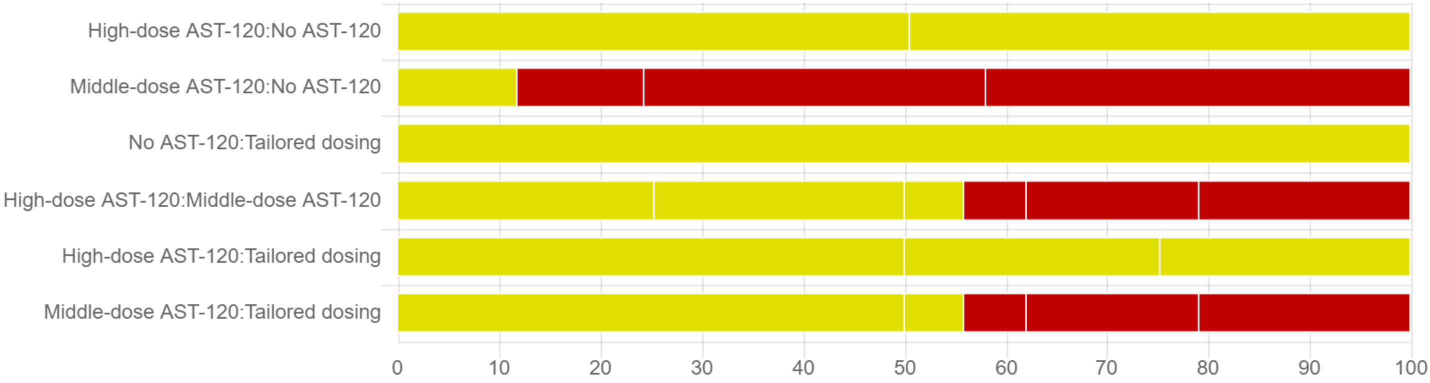

Composite renal outcome

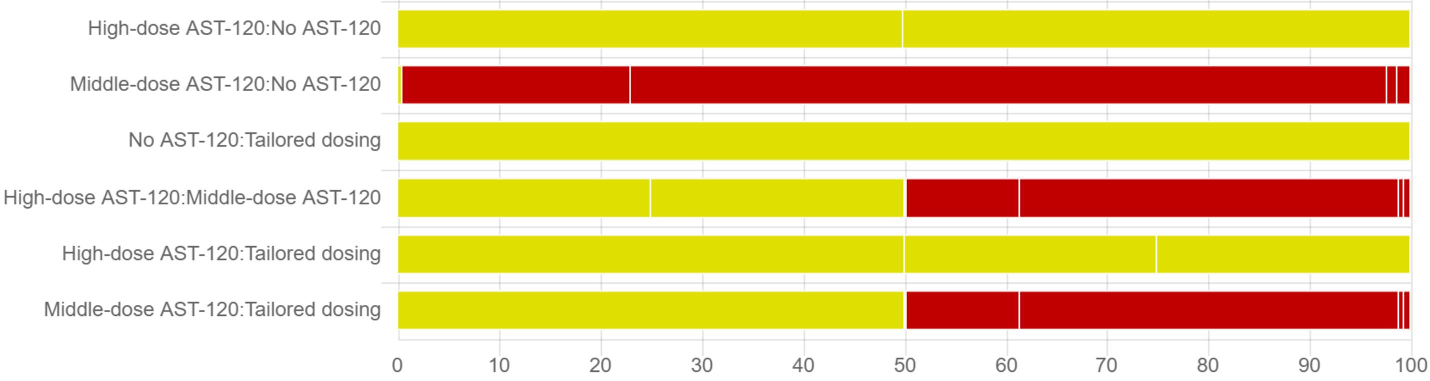

serum creatinine slope

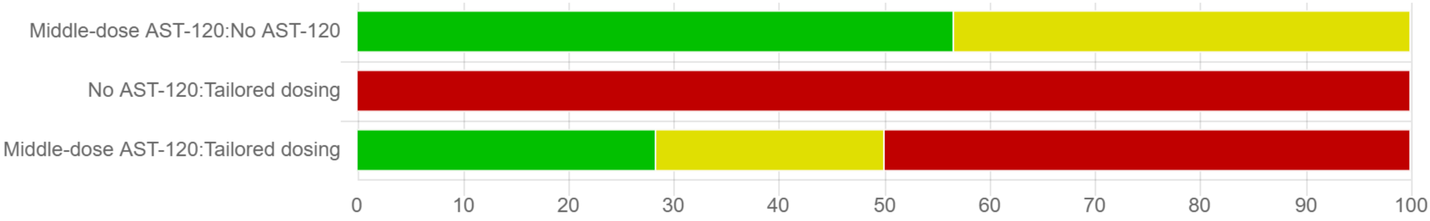

Supplementary File 2
Quality assessment (CINeMA approach)

Creatinine clearance

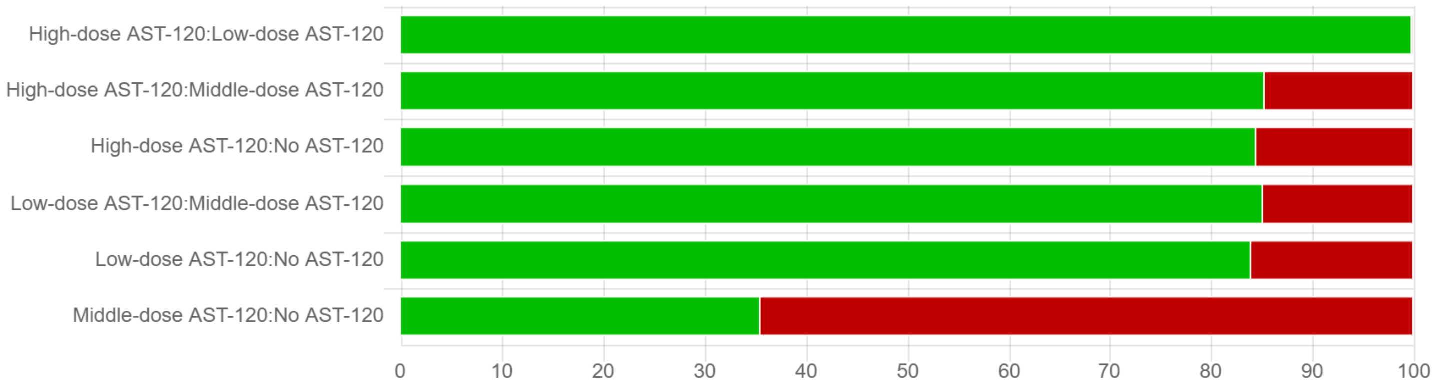

Indoxyl sulfate change

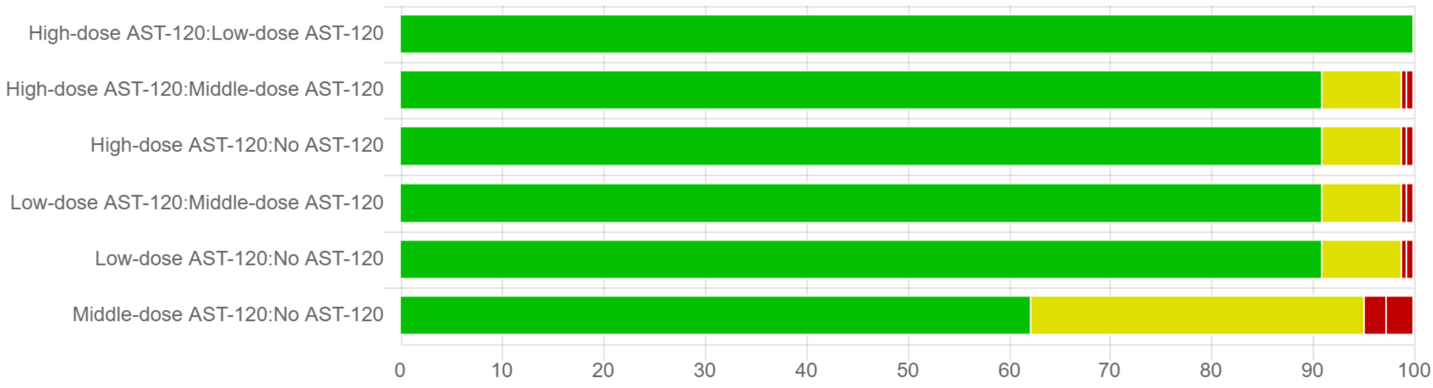

Urinary protein change

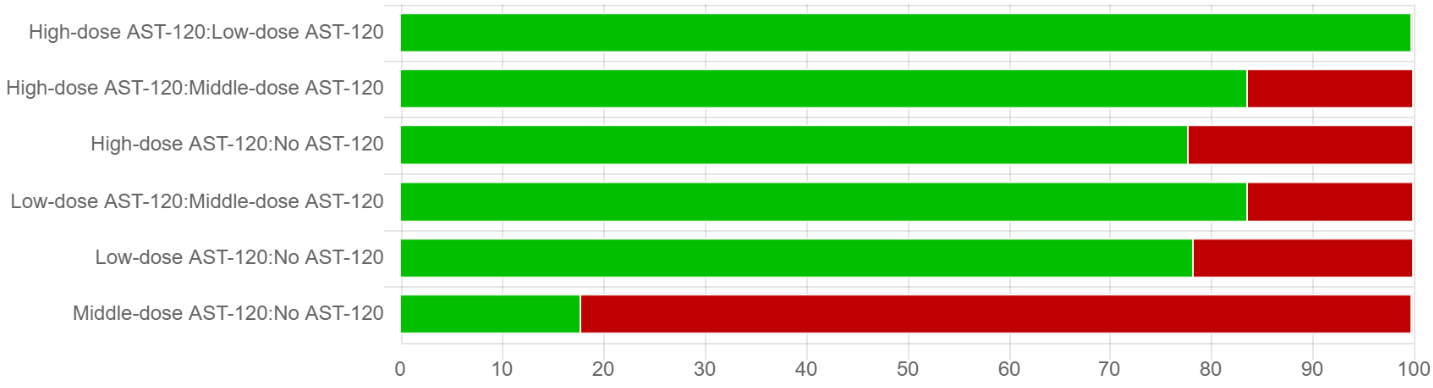

Supplementary File 3 to 4

Outcome of end stage renal disease

Supplementary File 3

Forest plot of direct evidence on end stage renal disease

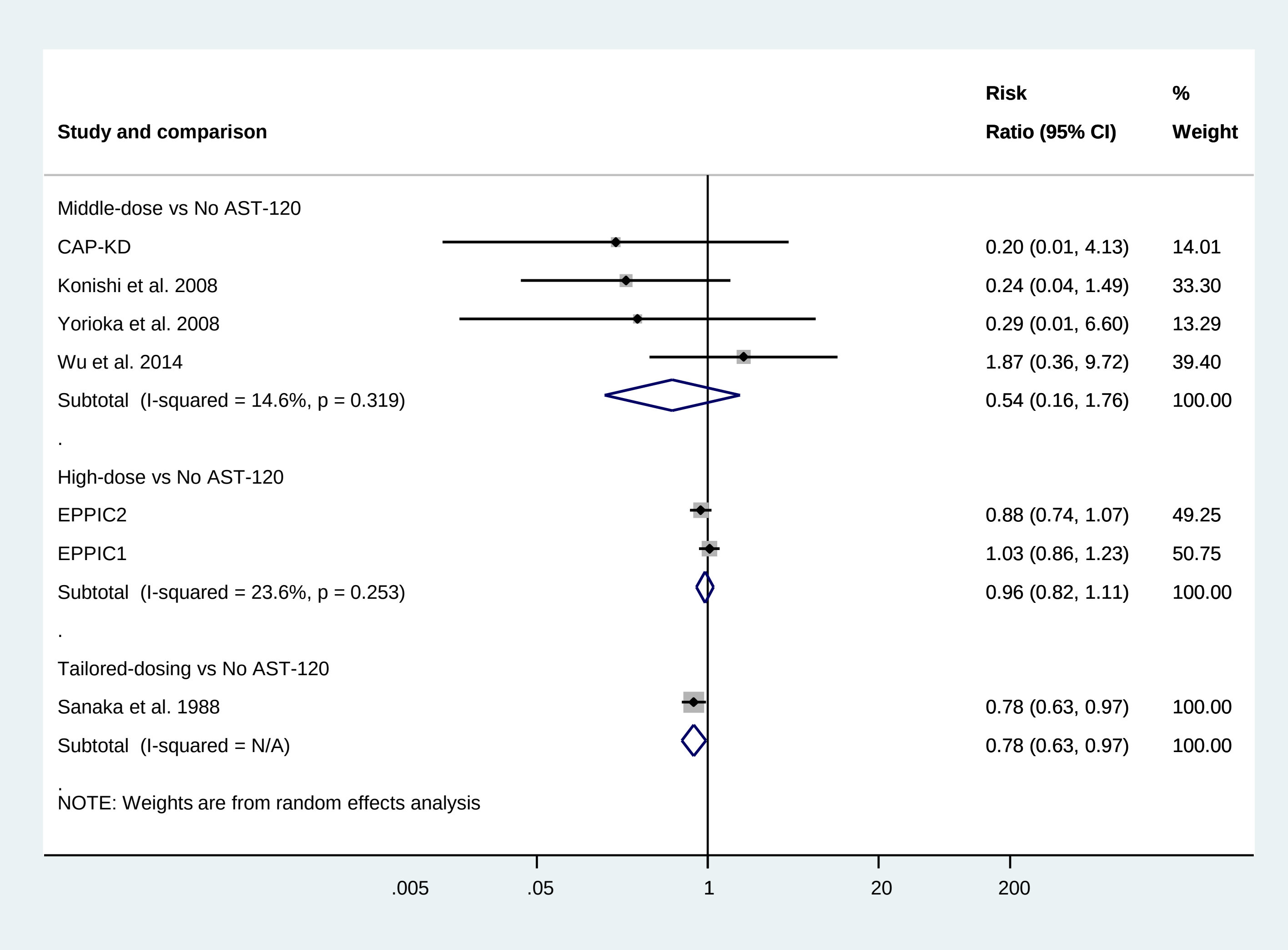

Supplementary File 4

Publication bias in network meta-analysis of end stage renal disease

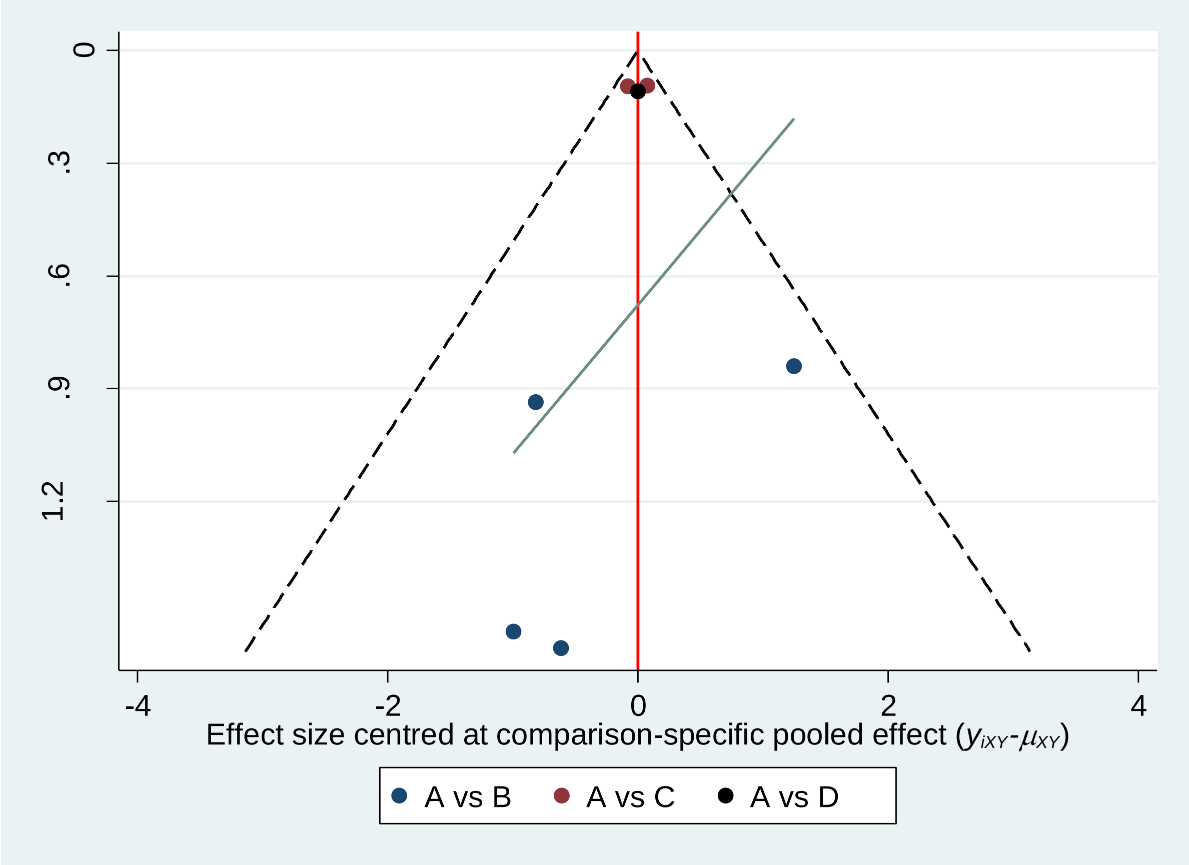

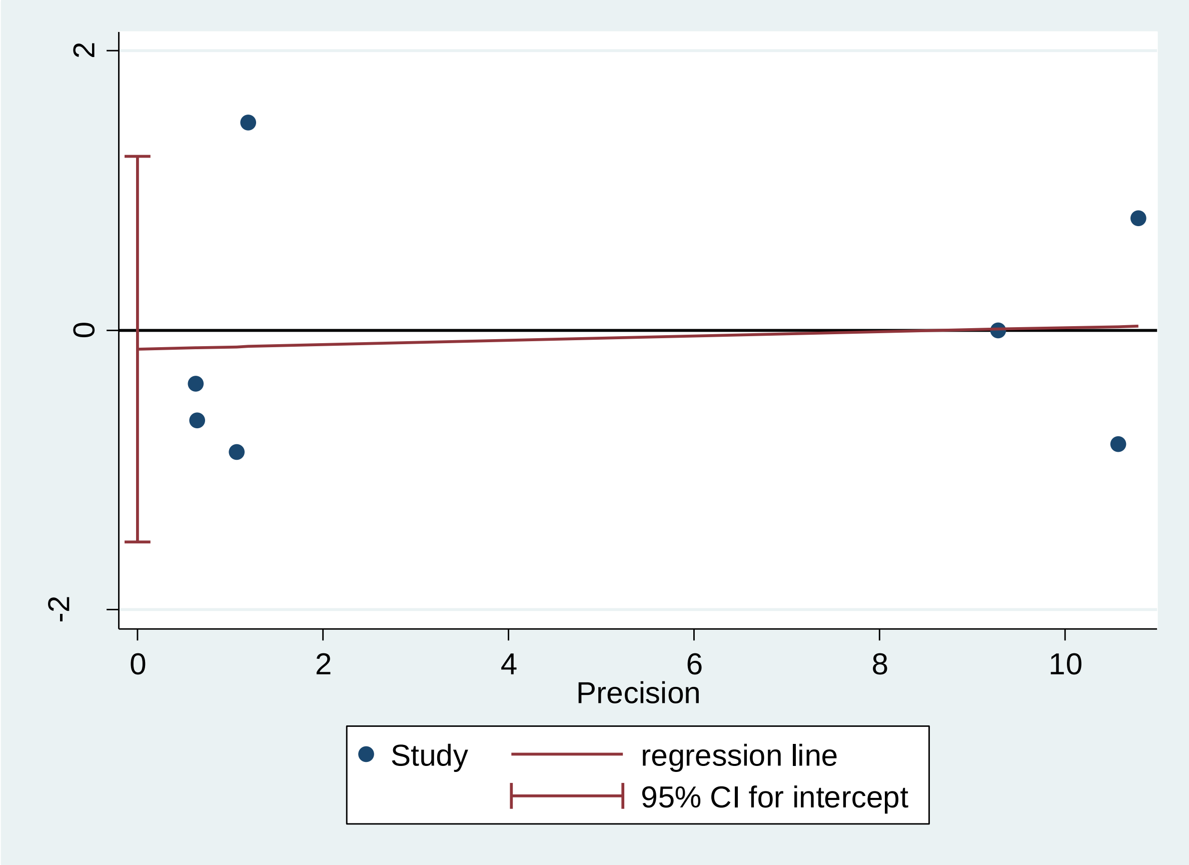

Supplementary File 5 to 6

Outcome of composite renal outcome

Supplementary File 5

Forest plot of direct evidence on composite renal outcome

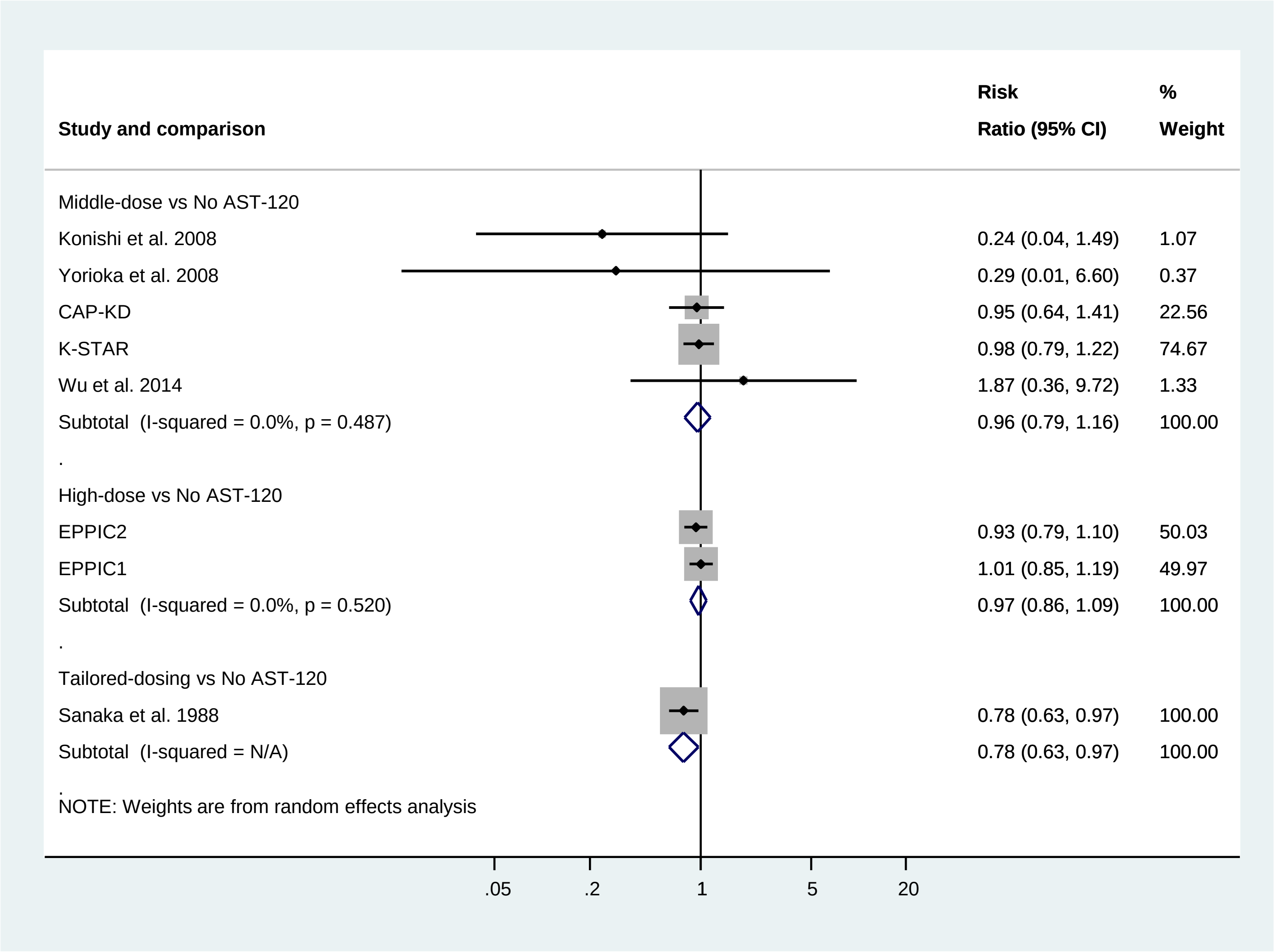

Supplementary File 6

Publication bias in network meta-analysis of composite renal outcome

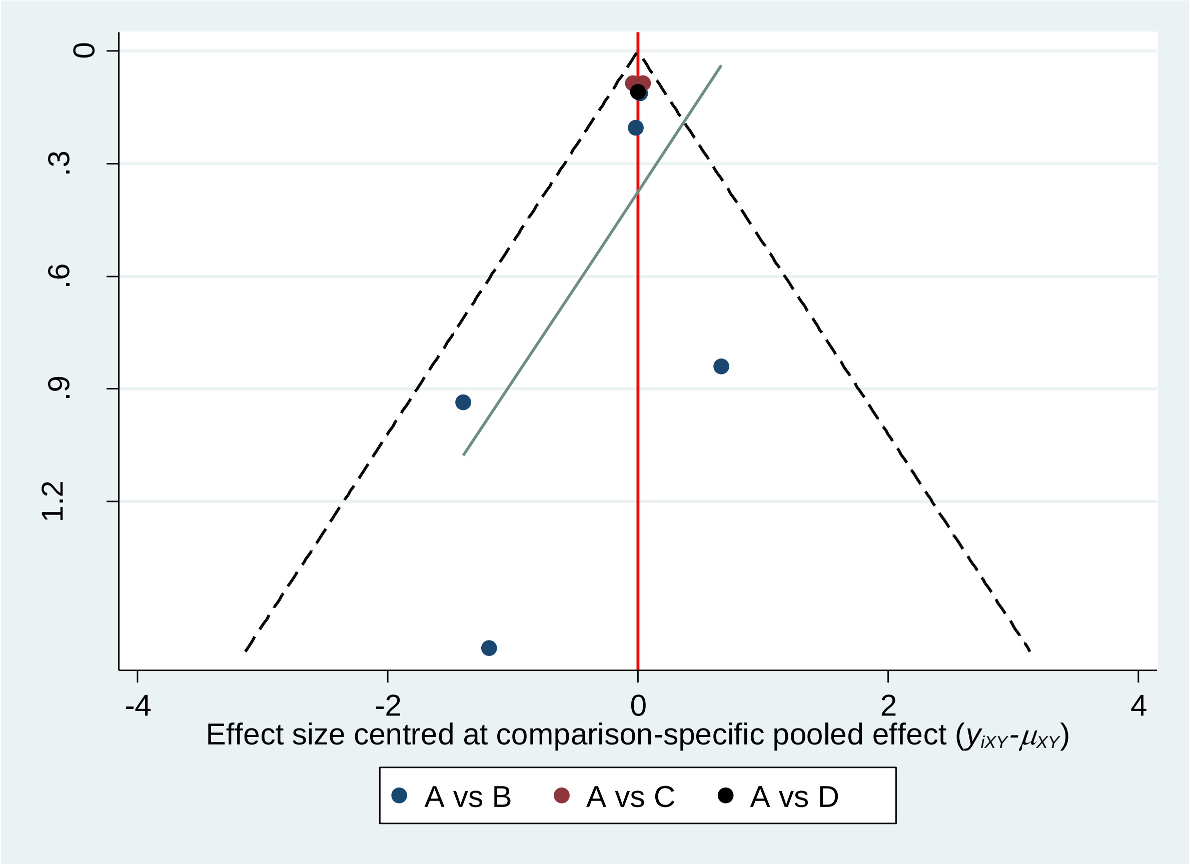

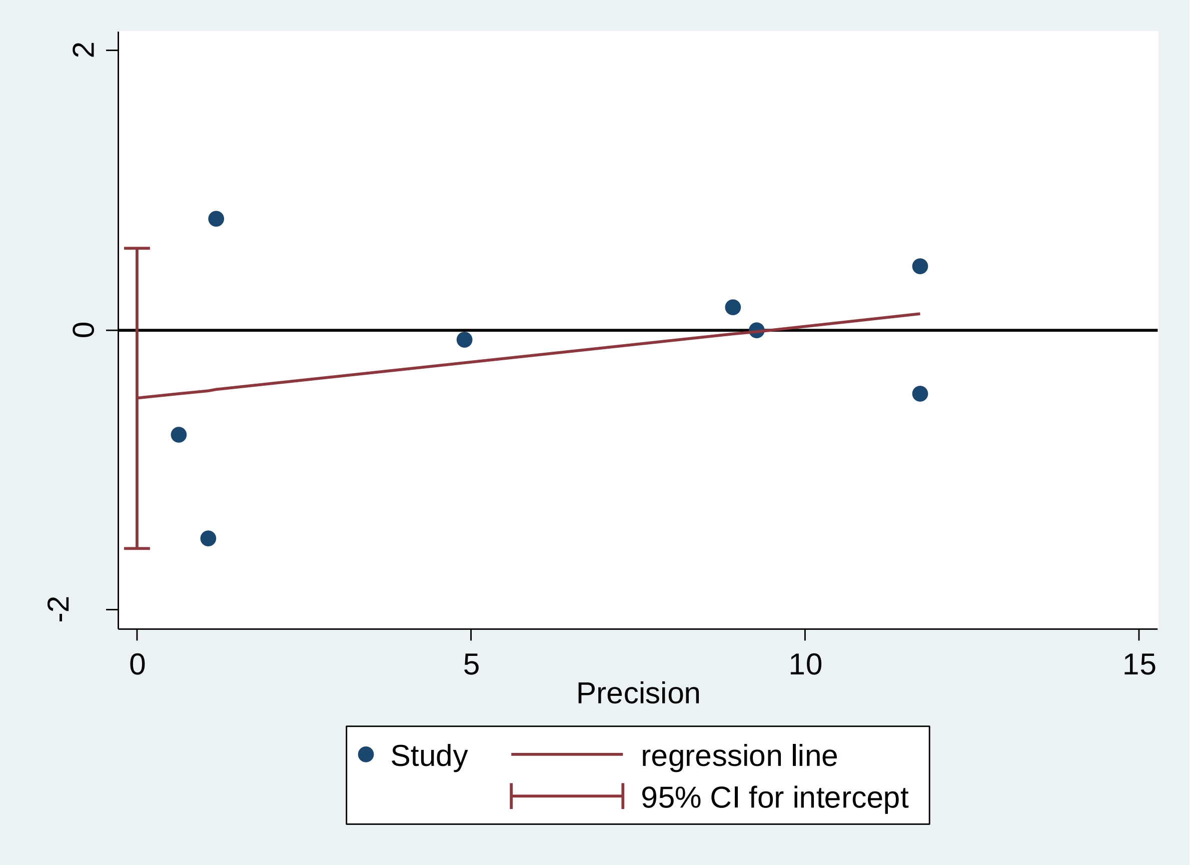

Supplement: Supplementary file 1 [file DataSheet1.pdf]
